# Triazine-Modified Color-Responsive Triarylboron/Acridine Fluorescent Probe with Multi-Channel Charge Transfer for Highly Sensitive Fluoride Ion Detection

**DOI:** 10.3390/molecules30040879

**Published:** 2025-02-14

**Authors:** Lei Tang, Jiaoyun Wang, Yuan Liu

**Affiliations:** School of Chemical Engineering and Light Industry, Guangdong University of Technology, Guangzhou 510006, China; tangleiTL0128@163.com (L.T.); jiaoyunwang2022@163.com (J.W.)

**Keywords:** detection of fluoride ions, triazine-modified, multiple charge-transfer states, color response

## Abstract

A novel fluoride ion fluorescent probe is designed by introducing the strong electron-withdrawing triazine groups into the triarylboron/acridine conjugation system. The A-D-A′ molecular configuration endows this molecule with multiple charge-transfer channels; upon reaction with F^−^, the triazine groups act as primary acceptors within the molecule, facilitating charge transfer between the acridine units and the triazine groups. During fluoride ion detection, changes in the triarylboron moiety lead to a significant bathochromic-shift in fluorescence emission from green to yellow. Theoretical calculations attribute this phenomenon to a reduction in the molecular *S*_1_ state energy level upon fluorination, resulting in a pronounced visible color change and chromogenic response during detection. Based on fluorescence intensity changes with varying degrees of F^−^ coordination, a detection limit as low as 10^−7^ M was determined for TB-1DMAc-2TRZ, demonstrating the high sensitivity of this probe.

## 1. Introduction

With the advancement of industrialization, substantial emissions of heavy metals and anions have begun to pose serious hazards to human living environments. Fluorine, characterized by its high electronegativity, non-metallic nature, small ionic radius, and high charge density, plays a significant role in various fields, including human health, medicine, chemistry, and industrial production [1,2,3]. Adequate fluoride intake can promote the growth of teeth and bones, effectively preventing cavities and treating osteoporosis [4,5,6,7]. However, prolonged exposure to excessive fluoride can result in a range of health issues, including Alzheimer’s disease, renal disorders, gastric cancer, and skeletal diseases [4,6,8]. Fluoride contamination has severely threatened several nations, placing over 200 million people globally at risk of fluoride toxicity [9,10]. To ensure human health, the U.S. Environmental Protection Agency (EPA) recommends that the fluoride concentration in drinking water does not exceed 2 ppm [8,11]. Consequently, in recent years, there has been considerable attention directed towards the detection of fluoride ions (F^−^) by researchers and the public alike [12,13,14,15,16,17,18,19,20,21].

Traditional fluoride ion detection methods typically require high-precision equipment and must be conducted under specific conditions. Additionally, these methods involve extensive data collection and complex calculations, resulting in relatively high measurement errors. Consequently, they have not been widely adopted in routine applications [22,23,24,25,26]. Currently, the simplest method for detecting fluoride ions is the use of fluorescent probes. In this process, the binding of the probe molecules to fluoride ions induces changes in their chemical structure, leading to alterations in the system’s absorption or fluorescence properties. These changes can be quantitatively measured using instruments or, in some cases, even observed with the naked eye. Therefore, fluorescent chemical sensors demonstrate significant potential for practical applications in fluoride detection [27]. Among the numerous methods for detecting F^−^, organic boron fluorescent probes stand out due to their advantages of high sensitivity, low detection limits, and excellent selectivity [28,29,30,31]. Currently, several types of fluorescent probes based on organic boron reactive sites have been developed, typically utilizing triarylboron as the reactive moiety. Triarylboron-based molecules possess unique electronic properties, with vacant *p*-orbitals in their periphery, allowing them to effectively accept excess electron pairs and function as excellent electron acceptors. These probes find widespread applications in organic light-emitting diodes (OLEDs), nonlinear optical materials (NLOMs), and fluoride ion detection [32,33,34,35,36,37,38,39]. Research indicates that steric hindrance between aryl substituents provides protection for the boron center, preventing interference from air and water and enhancing its stability. Moreover, the cage-like structure formed by triaryl groups can also shield the boron center from bulky anions, thereby improving the probe’s selectivity and resistance to interference when detecting F^−^ [40,41,42,43,44,45].

Various boron-based fluorescent molecules have been developed and have demonstrated excellent performance in fluoride ion detection [46,47,48,49,50]. However, issues such as detection limits (DLs) and visualization still require improvement [24,46]. In our previous study, we designed a triarylboron/acridine hybrid with additional phenyl groups, and the emission color blue-shifted from green to deep-blue and near-ultraviolet during detection [30]. To make the visualization easier to follow, it is necessary to adjust the emission color of the probe so that it is red-shifted within the visible region. In this study, we selected triarylboron derivatives and triazine moieties as electron-withdrawing units, and 9,9-dimethyl-9,10-dihydroacridine (DMAc) as an electron-donating unit, to design and synthesize a novel A-D-A′-type fluorescent probe, namely TB-1DMAc-2TRZ. The DMAc unit acts as a bridging element and simultaneously functions as a shared donor unit within the molecule. Triazine moieties, known for their rigid structure, were employed to further extend the conjugation system of the donor units [51,52]. The triazine group serves as a primary intramolecular acceptor, with charge transfer primarily occurring between DMAc and the triazine group. Triphenylboron functions both as a secondary intramolecular acceptor and as an active center for B-F reactions. During the detection of fluoride ions, the electronic structure of triphenylboron undergoes changes that hinder charge transfer between the donor unit and triphenylboron. Concurrently, the charge transfer between DMAc and the triazine group is further enhanced [53,54]. As a result, a certain degree of red-shift occurs in the fluorescence emission peak, which macroscopically manifests as a transition in fluorescence color from green to yellow. This study offers a valuable approach for creating color-responsive fluorescent probe molecules with high sensitivity and low detection limits.

## 2. Results and Discussion

### 2.1. Synthesis and Characterization

**Figure 1:**
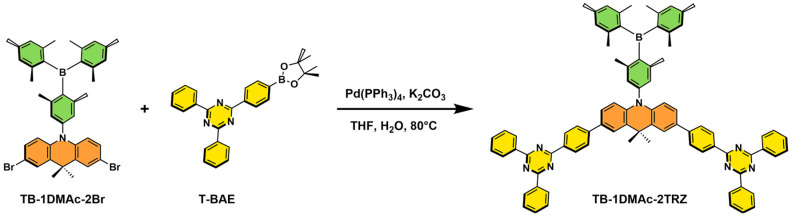
The synthesis route and chemical structure of TB-1DMAc-2TRZ.

The synthesis route and chemical structure of TB-1DMAc-2TRZ are depicted in Figure 1. Under an argon atmosphere, TB-1DMAc-2Br (0.72 g, 1.00 mmol), 2,4-diphenyl-6-[4-(4,4,5,5-tetramethyl-1,3,2-dioxaborolan-2-yl)phenyl]-1,3,5-triazine (1.31 g, 3.00 mmol), tetrakis (triphenylphosphine)palladium (92 mg, 0.08 mmol), and potassium carbonate (0.66 g, 4.8 mmol) were added to the device; then, 15 mL of THF and 5 mL of H_2_O were added to dissolve these, and the reaction solution was refluxed at 80 °C for 48 h. The resulting mixture was poured into water and extracted with dichloromethane. The combined organic layer was washed with water, and dried over Na_2_SO_4_. After removing the solvent under reduced pressure, the residue was purified with a silica gel column, using n-hexane/dichloromethane (6:1, *v*/*v*) as the eluent, to obtain the final products, followed by further recrystallization from a mixed solution of n-hexane/dichloromethane with a yield of 35% for 10-(4-(dimethicylboronalkyl)-3,5-dimethylphenyl)-9,9-dimethyl-2,7-triazine-9,10-dihydroacridine (TB-1DMAc-2TRZ) (0.42 g, green powder). ^1^H NMR (400 MHz, Chloroform-*d*) *δ* 8.86–8.79 (m, 12H), 7.86 (d, *J* = 2.1 Hz, 2H), 7.81 (d, *J* = 8.4 Hz, 4H), 7.61 (q, *J* = 6.8, 6.1 Hz, 12H), 7.42 (dd, *J* = 8.5, 2.1 Hz, 2H), 6.99 (s, 2H), 6.86 (s, 2H), 6.82 (s, 2H), 6.55 (d, *J* = 8.6 Hz, 2H), 2.32 (s, 6H), 2.19 (s, 6H), 2.15 (s, 6H), 2.08 (s, 6H), 1.89 (s, 6H). ^13^C NMR (100 MHz, Chloroform-*d*) *δ* 171.67, 171.59, 145.33, 143.73, 141.03, 140.56, 139.90, 136.49, 134.40, 132.70, 132.56, 130.53, 129.64, 129.09, 128.75, 126.62, 125.59, 124.48, 114.95, 36.52, 31.99, 23.21, 23.16, 23.09, 21.44. MALDI-TOF MS: *m*/*z* calculated for C_83_H_71_BN_7_^+^, 1176.587, found: 1176.916. Detailed synthetic routes and structural characterization of the remaining materials are given in the Supplementary Materials (Schemes S1 and S2, Figures S1–S9).

TB-1DMAc-2TRZ dissolves in typical organic solvents like dichloromethane and tetrahydrofuran. The TGA curve of TB-1DMAc-2TRZ, shown in Figure S10a, indicates a minimal weight loss of only 5% when the temperature reaches 432 °C. Additionally, the DSC curve in Figure S10b reveals a high glass transition temperature of 206 °C for TB-1DMAc-2TRZ. Both test results demonstrate the excellent thermal stability of TB-1DMAc-2TRZ, which is attributed to the modification with triazine moieties, which extend the conjugated system of the donor unit and enhance the molecular rigidity. The thermal data of TB-1DMAc-2TRZ are summarized in Table 1.

### 2.2. Photophysical Properties

**Figure 2:**
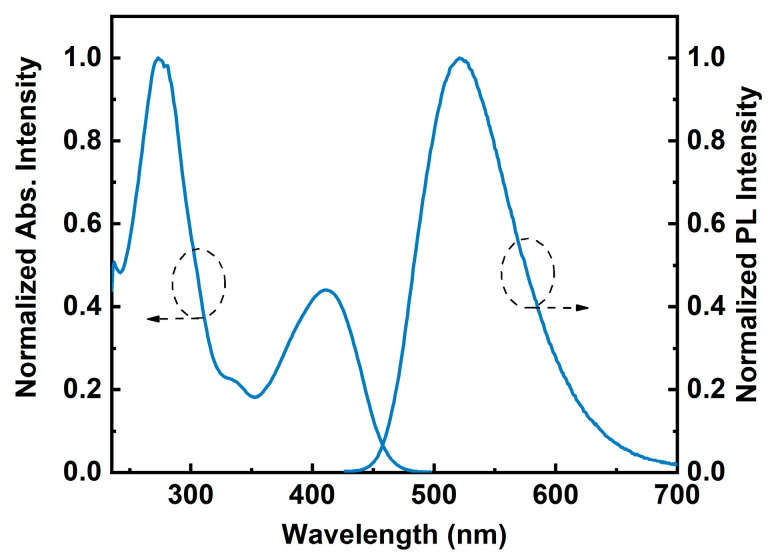
UV-vis absorption and emission spectra of TB-1DMAc-2TRZ in THF at a concentration of 10^−5^ M.

In a THF solution (10^−5^ M), the UV-visible absorption and fluorescence emission spectra of TB-1DMAc-2TRZ were recorded and plotted, as shown in Figure 2. The absorption peaks in the range of 250–320 nm originate from the local π − π* transitions of the triarylboryl unit and the DMAc-2TRZ unit, while the absorption band at 350–470 nm is mainly attributed to intramolecular charge transfer (ICT) from the DMAc donor unit to the TRZ acceptor unit. The fluorescence emission spectrum reveals a strong green emission peak at 522 nm for the TB-1DMAc-2TRZ. The position of this emission peak is intermediate between those of TB-1DMAc and TB-1DMAc-2Ph, indicating that the electron-donating ability of DMAc-2TRZ lies between that of DMAc and DMAc-2Ph [15]. This phenomenon is attributed to the reduced electron-donating ability of DMAc-2Ph, due to the modification of the triazine groups. The photophysical data of TB-1DMAc-2TRZ are summarized in Table 1.

### 2.3. Detection of Fluoride Ions

**Figure 3:**
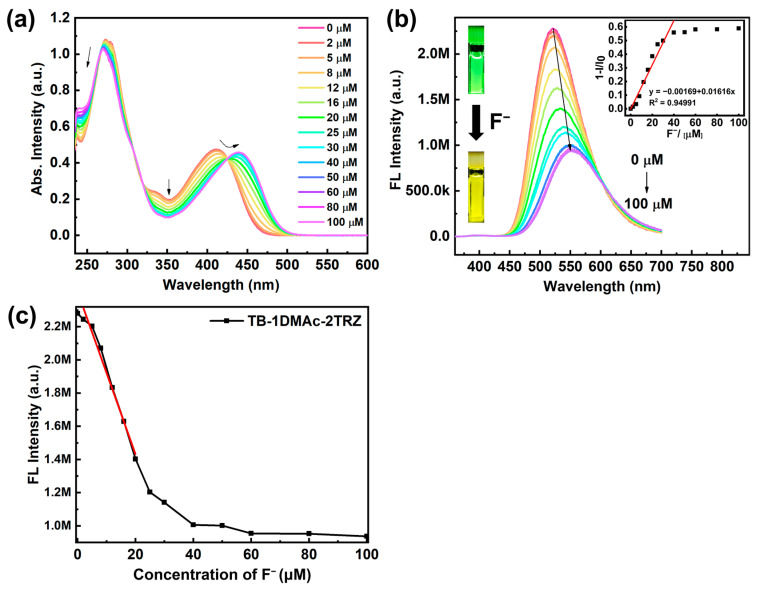
In THF solvent, incremental additions of TBAF to a 10 μM probe solution were monitored. (**a**) UV-visible absorption spectra, (**b**) steady-state fluorescence emission spectra, and (**c**) fluorescence spectrum with linear fitting of fluorescence intensity vs. F^−^ concentration. The insets of panel (**b**) show a plot of the change in response intensity vs. F^−^ concentration, along with photographs taken under UV light before and after the addition of F^−^. The red line segment in the inset of panel (**b**) and panel (**c**) both represent the trend line of the data within the linear range.

Triarylboron possesses unique electronic properties, due to its vacant *p*-orbitals in the periphery, which are capable of accepting excess electron pairs and reacting with fluoride ions. Herein, using THF as the solvent, we prepared solutions of the fluorescent probe and tetrabutylammonium fluoride (TBAF) as a fluoride source, and investigated TB-1DMAc-2TRZ’s response to F^−^ through UV-vis absorption and fluorescence spectra. The UV-vis absorption titration experiment results of TB-1DMAc-2TRZ are shown in Figure 3a. During titration, the absorption peak intensity at 410 nm gradually decreases, while that at 450 nm increases, reaching an equivalence point, which indicates fluoride binding to the boron center to form a stable compound. The ^19^F NMR spectrum shown in Figure S11 also indicates that the fluoride ions are effectively coordinated with the boron center of TB-1DMAc-2TRZ. Fluorescence titration results, as shown in Figure 3b, show a gradual red-shift of the probe’s emission peak from 522 nm to 550 nm with increasing fluoride ion concentration. Upon addition of 40 μM fluoride ions, the fluorescence intensity of the probe system reduces to approximately one-fourth of its original intensity. During fluorescence titration, illumination with a 365 nm UV lamp reveals a noticeable transition of the probe’s emission from green to yellow, which is attributed to alterations in the electronic structure of triarylboron due to the B-F reaction. This enhances the charge transfer between the donor unit DMAc and the triazine moiety, leading to a noticeable red-shift in fluorescence and enhanced visual detectability. Within the range of 0–30 μM fluoride ion additions, the fluorescence emission intensity of the probe system exhibits a strong linear correlation with fluoride ion concentration, as illustrated in Figure 3c. Based on the results of the fluorescence titration, the detection limit of TB-1DMAc-2TRZ is approximately 3.12 × 10^−7^ M. When compared to other fluorescent probes for fluoride ion detection reported in the literature, this probe system demonstrates a high level of sensitivity. The comparative data can be found in Table S1. Modification of the probe with electron-withdrawing triazine moieties on the DMAc donor unit enables significant visible light color change before and after fluoridation, enhancing its visual observation capabilities. The binding constant between TB-1DMAc-2TRZ and F^−^, as determined from the fitted Benesi–Hildebrand plots, is 1.80 × 10^4^ M^−1^ (Figure S12), which is nearly one order of magnitude higher than that between TB-1DMAc-2Ph and F^−^ [30].

### 2.4. Theoretical Calculations

**Figure 4:**
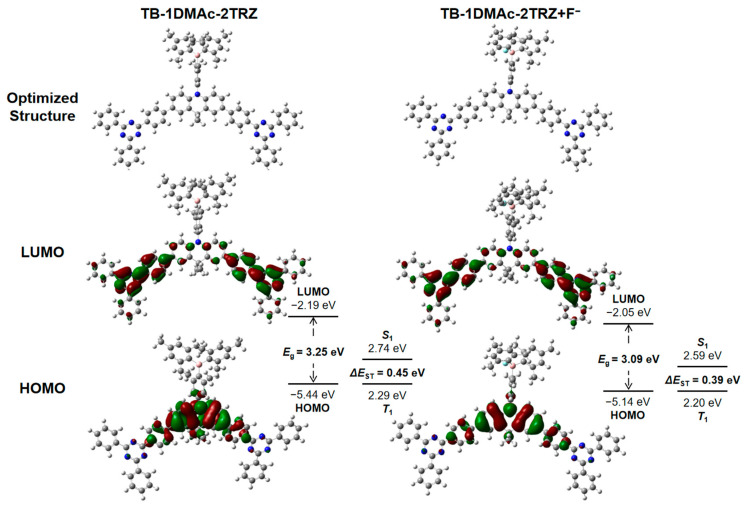
Optimized structures and frontier molecular orbitals (FMOs) of TB-1DMAc-2TRZ before and after fluorination, along with computed HOMO/LUMO energy levels, band gap (*E*_g_s), and *S*_1_/*T*_1_ energy levels. For the adducts, methylamine is used in the place of butylamine to simplify the calculations.

To better validate the phenomenon of emission peak red-shifting upon fluoride ion binding, and to comprehensively understand the B-F binding effect, theoretical calculations were performed on TB-1DMAc-2TRZ compounds before and after fluoridation. The frontier orbital distributions of TB-1DMAc-2TRZ before and after fluoridation are shown in Figure 4. The LUMO energy level of TB-1DMAc-2TRZ is predominantly distributed on the acceptor unit, 2Ph-2TRZ, while the HOMO energy level is primarily distributed on the donor unit, DMAc. This distribution arises from the strong electron-withdrawing capability of the triazine moiety, making it the primary acceptor in this probe system. Additionally, overlapping of HOMO and LUMO levels at the phenyl group of the donor unit indicates significant conjugation between the donor unit and the triazine moiety.

As the B-F reaction progresses, changes occur in the electronic structure of triarylboron, shifting the charge transfer from the original triarylboron to the donor unit, which transforms into charge transfer from the triazine moiety to the donor unit. This results in the fluorescence emission of the probe system primarily originating from the unit composed of the donor unit and the primary acceptor, the triazine moiety. According to DFT theoretical calculations, the *S*_1_ state of the molecule after fluoridation is lower than that before fluoridation, which is the main factor causing the red-shift in the fluorescence emission peak. Upon addition of an F^−^ source, macroscopically, the probe system exhibits a transition from green to yellow emission, demonstrating significant visible color change and effective visualization. The theoretical calculation results are shown in Table 1.

### 2.5. Photostability, Repeatability, Selectivity, and Anti-Interference Analysis

**Figure 5:**
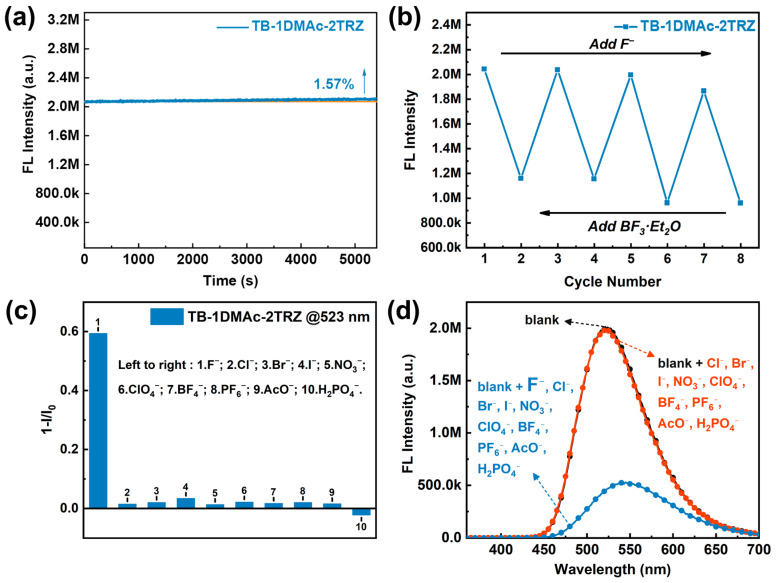
(**a**) Photobleaching stability of TB-1DMAc-2TRZ in THF, (**b**) repeatability of TB-1DMAc-2TRZ, (**c**) a quantitative histogram of the fluorescence response of TB-1DMAc-2TRZ to various anions (50 μM of Bu_4_N^+^), and (**d**) anti-interference of TB-1DMAc-2TRZ with different anions (150 μM of Bu_4_N^+^).

To ensure the reliability and accuracy of the experiment, the photostability of the TB-1DMAc-2TRZ probe was analyzed, as shown in Figure 5a. After 1.5 h of photobleaching, the fluorescence intensity of TB-1DMAc-2TRZ not only remained stable, but also actually increased by approximately 1.57%, indicating the probe’s excellent stability. The modification of the triazine group on DMAc-2Ph extends the conjugated system of the donor unit and enhances the rigidity of the molecule, thereby maintaining a high level of photostability in the fluorescence probe.

To meet environmental protection requirements, repetitive analysis experiments were designed. By alternately adding a TBAF solution and a stronger fluoride ion scavenger, a BF_3_•Et_2_O solution, the fluorescence emission of the probe system transitioned from green to yellow and reverted to green upon repetition for four times. This consistent behavior was confirmed by measuring the fluorescence intensity changes throughout the process, as depicted in Figure 5b. The fluorescence emission intensity showed no significant variation over the four tests, due to the reversible reaction between the probe’s detection group and the fluoride ions. When a stronger electron-withdrawing fluoride ion scavenger was introduced, the reaction reversed, restoring the fluorescence intensity to its initial state. Therefore, the TB-1DMAc-2TRZ probe system exhibits excellent reproducibility, making it suitable for repeated use in practical applications, and promoting sustainable ecological development.

To evaluate the selectivity of TB-1DMAc-2TRZ, a series of anions, namely F^−^, Cl^−^, Br^−^, I^−^, NO_3_^−^, ClO_4_^−^, BF_4_^−^, PF_6_^−^, AcO^−^, and H_2_PO_4_^−^, were introduced into the probe solution, and their fluorescence emission spectra were recorded, as shown in Figure 5c. It was observed that the fluorescence emission of TB-1DMAc-2TRZ at 523 nm changed significantly only upon the addition of F^−^, while other anions did not induce such a response. Interference resistance is another critical parameter for chemical sensors. An interference analysis of TB-1DMAc-2TRZ was conducted; the results of this are displayed in Figure 5d. Despite the presence of large quantities of Cl^−^, Br^−^, I^−^, NO_3_^−^, ClO_4_^−^, BF_4_^−^, PF_6_^−^, AcO^−^, and H_2_PO_4_^−^, the probe system maintained high sensitivity to F^−^ without a significant loss of performance. Only with the addition of F^−^ did the probe system show a transition from green to yellow under UV light at 365 nm, with a gradual decrease in fluorescence intensity observed by eye. Fluorescence emission spectra recorded using a fluorometer confirmed that F^−^ caused a red-shift in the emission peak and reduced the fluorescence intensity to about one-quarter of its original value. The excellent selectivity and interference resistance of the TB-1DMAc-2TRZ probe towards F^−^ are attributed to the cage-like structure formed by the methyl groups on the triarylboron unit, which provides spatial protection to the central boron atom. Larger anions are effectively excluded, while the smaller F^−^ can access the boron center and interact with it. Thus, the TB-1DMAc-2TRZ probe demonstrates outstanding selectivity and resistance to interference during F^−^ detection, making it an ideal probe for fluoride ion detection.

### 2.6. Visual Applications for Fluoride Ion Detection

**Figure 6:**
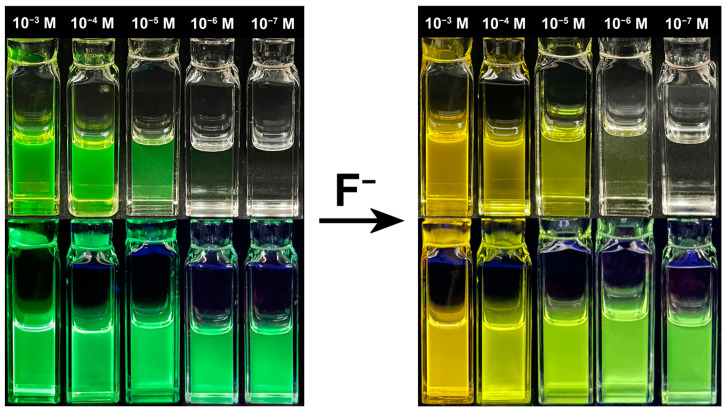
The TB-1DMAc-2TRZ-based THF solution test for visual F^−^ detection with various concentrations, under normal light and 365 nm UV irradiation.

Leveraging the color tunability of TB-1DMAc-2TRZ in the visible light range, the color changes of THF solutions containing different concentrations of the fluorescent probe were recorded under normal light and 365 nm UV illumination for fluoride ion detection. As shown in Figure 6, the addition of fluoride ions at a concentration of 10^−5^ M resulted in a noticeable color change from green to yellow under both normal and UV light. Notably, even at a probe solution concentration as low as 10^−7^ M, significant color changes before and after the addition of fluoride ions could still be detected under UV light. This indicates that the probe solution has considerable potential for developing a high-contrast colorimetric method for fluoride detection.

## 3. Conclusions

In summary, a novel A-D-A′-structured organic boron fluorescent probe, namely TB-1DMAc-2TRZ, was constructed by modifying the donor unit with strongly electron-withdrawing triazine groups. During the B-F reaction, TB-1DMAc-2TRZ undergoes a transformation from homolytic hybridization to heterolytic hybridization, facilitating multiple charge-transfer channels within the molecule; the primary acceptor unit shifts from triarylboron to a triazine moiety, thus enabling charge transfer between the donor unit and triazine moiety. The triarylboron, at this stage, acts as a secondary acceptor within the molecule and serves as the focal point of the B-F reaction. Retaining the characteristic triarylboron structure allows TB-1DMAc-2TRZ to function effectively as an F^−^ detection probe, reacting with fluoride ions. Prior to fluoridation, TB-1DMAc-2TRZ exhibits green emission in tetrahydrofuran solution. Post-fluoridation, theoretical calculations indicate a lowering of the molecule’s S1 state energy level, prompting a transition in the probe’s fluorescence from green to yellow emission, which demonstrates a robust color response. Furthermore, titration analysis of the fluorescence spectra as a function of varying fluoride ion concentrations revealed that TB-1DMAc-2TRZ has a detection limit of approximately 10^−7^ M, demonstrating its high sensitivity. This study highlights that organic boron molecules modified with triazine groups can serve as color-responsive fluorescent probes, offering a feasible design strategy for the structural development of fluoride probes and the enhancement of probe sensitivity and visualization effects.

## Figures and Tables

**Table 1:** Photophysical, thermal, and electrochemical characteristics of TB-1DMAc-2TRZ.

Compound	*λ*_abs_ ^1^ [nm]	*λ*_em_ ^1^ [nm]	*T*_d_ ^2^ [°C]	*T*_g_ ^3^ [°C]	*E*_S_/*E*_T_ ^4^ [eV]	*ΔE*_ST_ ^5^ [eV]	HOMO ^6^/LUMO ^7^ [eV]	*E*_g_ ^8^ [eV]
TB-1DMAc-2TRZ	272/411	522	432	206	2.74/2.29	0.45	−5.44/−2.19	3.25

^1^ Measured at room temperature in tetrahydrofuran (10^−5^ M). ^2^ The temperature at which the material lost 5% of its weight. ^3^ Determined from the 2nd heating scan. ^4^ Estimated from time-resolved photo-luminescence spectra of pure films. ^5^ Derived from *E*_S_–*E*_T_ calculations. ^6^ Calculated from the onset potential point of the oxidation curve. ^7^ Inferred from HOMO and optical *E*_g_. ^8^ Derived from the absorption edge wavelength.

## Data Availability

Data are available on request from the authors.

## Supplementary Materials

The following supporting information can be downloaded at https://www.mdpi.com/article/10.3390/molecules30040879/s1: Schemes S1 and S2: Synthetic routes of materials; Figures S1, S4, and S7: ^1^H NMR spectra of materials; Figures S2, S5, and S8: ^13^C NMR spectra of materials; Figures S3 and S6: HRMS spectra of materials; Figure S9: MS spectra of material; Figure S10: TGA and DSC of material; Figure S11: ^19^F NMR spectra of TBAF and TB-1DMAc-2TRZ mixture after addition of TBAF; Figure S12: Benesi–Hildebrand plots for complexation of material with F^−^; Table S1: Comparison of TB-1DMAc-2TRZ with other reported F^−^ detection probes. Refs. [1,4,55,56,57,58,59,60,61,62,63,64] are cited in the Supplementary Materials.
