# Supplementary material for: Triazine-Modified Color-Responsive Triarylboron/Acridine Fluorescent Probe with Multi-Channel Charge Transfer for Highly Sensitive Fluoride Ion Detection
Source: Molecules. 2025 Feb 14;30(4):879. doi: 10.3390/molecules30040879 (PMC11858165; doi:10.3390/molecules30040879)
Supplement: Supplementary file 1 [file molecules-30-00879-s001.zip › molecules-3418461-supplementary.pdf]

SUPPLEMENTARY MATERIAL

Triazine-Modified Color-Responsive Triarylboron/Acridine Fluorescent Probe with Multi-Channel Charge Transfer for Highly Sensitive Fluoride Ion Detection

Lei Tang, Jiaoyun Wang and Yuan Liu

1. Experimental Section

1.1 Materials. All the reagents and solvents used for the synthesis or measurements were commercially available and used as received unless otherwise stated. Toluene was dried by calcium hydride.

1.2 Instrumental Methods. The ^{1}H NMR, ^{13}C NMR and ^{19}F NMR spectra were recorded by a Brucker ADVANCE III 400 MHz superconducting front-end instrument with chloroform-*d* as solvent and tetramethylsilane (TMS) as internal reference. High-resolution mass spectra (HRMS) of TB-1DMAc and TB-1DMAc-2Br were collected on a Bruker maxis UHR-TOF mass spectrometer in an ESI positive mode. MALDI-TOF mass spectra of TB-1DMAc-2TRZ was collected on a Bruker autoflex time-of-flight mass spectrometer. UV-vis absorption spectra were recorded on a Shimadzu UV-2700 recording spectrophotometer. Photoluminescence (PL) spectra were recorded on a HORIBA Fluoromax-4C-L spectrophotometer. Thermogravimetric Analysis (TGA) was performed on a NETZSCH STA409PC unit at a heating rate of 10 °C/min from 25 to 700 °C under N_2. Differential scanning calorimetry (DSC) was carried out on METTLER TOLEDO DSC3 at a heating rate of 10 °C/min from 25 to 350 °C for TB-1DMAc-2TRZ under N_2. The glass transition temperature (T_g) was determined from the second heating scan at a heating rate of 10 °C/min.

1.3 Theoretical Calculations. Theoretical simulations were performed using the Gaussian 09 program packages. The density functional theory (DFT) calculations at the B3LYP(D3)/def2-SVP level was used to optimize the ground state geometries of the investigated molecules. Time-dependent density functional theory (TD-DFT) calculations were performed at PBE0/def2-SVP according to the optimized ground state geometries.

1.4 Photochemical Stability. The photochemical stability of the representative compounds in THF solution was tested using a HORIBA Fluoromax-4C-L spectrophotometer with a time scan pattern and a 150 W Xenon lamp was employed as the light source. Typical irradiation time was 1.5 h.

1.5 Anion sensing studies. Stock solutions (1 mM) of probes was prepared in THF and the final concentrations were 10 μM by a 100x dilution of the stock solution. Solutions (0.1 and 1 mM) of the tetrabutylammonium salts of the respective anions were prepared in THF. The concentration of sensor compounds was kept constant throughout the titration process, while adding increasing amounts of anion to the sensor solution. Then the UV-vis and fluorescence emission spectra were recorded at room

temperature. The selectivity properties were explored towards Cl^-, Br^-, I^-, NO_3^-, ClO_4^-, BF_4^-, PF_6^-, AcO^- and $H_2PO_4^-$ (100 μM) by the sensor concentration in THF in the fluorescence spectra.

1.6 Determination of Detection Limit (DL). The detection limit of the sensor has been determined according to the following equations or functions:

$$S_b = \sqrt{\frac{\sum_{i=1}^{n}(x_i - \bar{x})^2}{n-1}} \quad (1)$$

$$S = \frac{\Delta I}{\Delta c} \quad (2)$$

$$DL = \frac{3S_b}{S} \quad (3)$$

The standard deviation (S_b) regarding present fluorophores and the instrument was determined by measuring the fluorescence intensities (x_i) in THF for more than 100 times, and calculating the corresponding average intensity ($\bar{x}$) firstly. By fitting the intensity data and the average intensity as obtained into equation (1), the value of the standard deviation (S_b) was obtained.

Then, F^- was added into the solution of relevant fluorophores with different concentrations, and then the fluorescence emission intensities were recorded (Fig. 3b). Corresponding variations in intensity (ΔI) and the F^- concentration (Δc) were calculated. By fitting the data into equation (2), S value for the present system was obtained.

Finally, with the values of S_b and S as determined, the DL for the present system was calculated according to equation (3).

2. Synthesis

(4-bromo-2,6-dimethylphenyl)dimesitylborane (**TB-1Br**), 10-(4-(dimesitylboranyl)-3,5-dimethylphenyl)-9,9-dimethyl-9,10-dihydroacridine (**TB-1DMAc**), 2,7-dibromo-10-(4-(dimesitylboranyl)-3,5-dimethylphenyl)-9,9-dimethyl-9,10-dihydroacridine (**TB-1DMAc-2Br**) were synthesized according to the literature methods [55-57].

Synthesis of TB-1Br

(4-bromo-2,6-dimethylphenyl)dimesitylborane (**TB-1Br**): Under Ar atmosphere, a hexane solution of *n*-BuLi (2.5 M, 4.4 mL, 11 mmol) at -78 °C was added dropwise to a solution of 5-bromo-2-iodo-

1,3-dimethylbenzene (3.11 g, 10 mmol) in dry Et_2O (20 mL). The reaction mixture was allowed to warm to 0 °C and stirred for 20 min. Then, fluorodimesitylborane (2.55 g, 9.5 mmol) in dissolved dry Et_2O (10 mL) was dropped into the mixture at -78 °C. The reaction mixture was warmed up to room temperature and stirred overnight. The mixture was filtered to give the product as a white solid (3.1 g, 75%). ^{1}H NMR (400 MHz, Chloroform-*d*) δ (ppm) 7.08 (s, 2H), 6.74 (s, 4H), 2.26 (s, 6H), 1.98 – 1.94 (m, 18H).

Synthesis of TB-1DMAc

10-(4-(dimesitylboranyl)-3,5-dimethylphenyl)-9,9-dimethyl-9,10-dihydroacridine (**TB-1DMAc**): To a mixture of TB-1Br (2.16 g, 5.00 mmol), 9,9-diphenyl-9,10-dihydroacridine (1.16 g, 5.50 mmol), $Pd(OAc)_2$ (56 mg, 0.26 mmol), $[(t\text{-Bu})_3PH]BF_4$ (220 mg, 0.76 mmol) and *t*-BuONa (1.73 g, 18.00 mmol) were added redistilled toluene (40 mL), and the solution was refluxed for overnight at 120 °C under argon. The resulting mixture was poured into water and extracted with dichloromethane. The combined organic layer was washed with water, and dried over Na_2SO_4. After removed the solvent under reduced pressure, the residue was purified with a silica gel column using *n*-hexane/dichloromethane (5:1, *v*/*v*) as the eluent to obtain the final products and further recrystallization from a mixed solution of *n*-hexane/dichloromethane with a yield of 68% for TB-1DMAc (1.90 g, white powder): ^{1}H NMR (400 MHz, Chloroform-*d*) δ (ppm) 7.44 (dd, J = 7.7, 1.6 Hz, 2H), 7.00 (m, 2H), 6.95 – 6.89 (m, 4H), 6.82 (s, 2H), 6.79 (s, 2H), 6.39 (dd, J = 8.1, 1.3 Hz, 2H), 2.31 (s, 6H), 2.13 (s, 6H), 2.09 (s, 6H), 2.05 (s, 6H), 1.68 (s, 6H). ^{13}C NMR (100 MHz, Chloroform-*d*) δ (ppm) 143.44, 141.49, 140.95, 140.85, 140.61, 139.76, 130.08, 129.95, 128.99, 128.93, 126.43, 125.19, 120.51, 114.14, 36.09, 31.24, 23.12, 23.02, 21.41. HRMS: *m*/*z* calculated for $C_{41}H_{45}BN^+$, 562.3645, found: 562.3646.

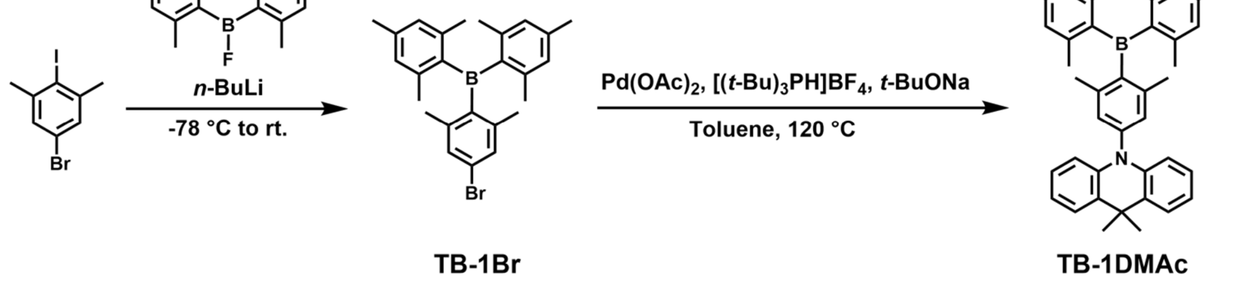

Scheme S1. Synthetic route of TB-1DMAc.

Synthesis of TB-1DMAc-2Br

2,7-dibromo-10-(4-(dimesitylboranyl)-3,5-dimethylphenyl)-9,9-dimethyl-9,10-dihydroacridine (**TB-1DMAc-2Br**): *N*-Bromosuccinimide (0.96 g, 5.40 mmol) dissolved in 12 mL of THF was slowly added to a solution of TB-1DMAc (1.01 g, 1.80 mmol) in 18 mL of THF. The reaction mixture was stirred at room temperature for 10 h under ambient atmosphere. The resulting mixture was poured into water and extracted with dichloromethane. The combined organic layer was washed with water, and dried over Na_2SO_4. After removed the solvent under reduced pressure, the residue was purified with a silica gel column using *n*-hexane/dichloromethane (8:1, *v*/*v*) as the eluent to obtain the final products and further recrystallization from a mixed solution of *n*-hexane/dichloromethane with a yield of 75% for TB-1DMAc-2Br (0.97 g, white powder). ^{1}H NMR (400 MHz, Chloroform-*d*) δ (ppm) 7.48 (d, J = 2.3 Hz, 2H), 7.09 (dd, J = 8.8, 2.3 Hz, 2H), 6.83 – 6.77 (m, 6H), 6.23 (d, J = 8.7 Hz, 2H), 2.29 (s, 6H), 2.08 (d, J = 5.3 Hz, 12H), 2.04 (s, 6H), 1.62 (s, 6H). ^{13}C NMR (100 MHz, Chloroform-*d*) δ (ppm) 143.77, 141.02, 140.62, 140.49, 139.94, 139.69, 131.69, 129.46, 129.38, 129.03, 129.01, 128.05, 116.00, 113.25, 36.39, 30.99, 23.12, 23.10, 23.02, 21.41. HRMS: *m*/*z* calculated for $C_{41}H_{43}BBr_2N^+$, 718.1855, found: 718.1859.

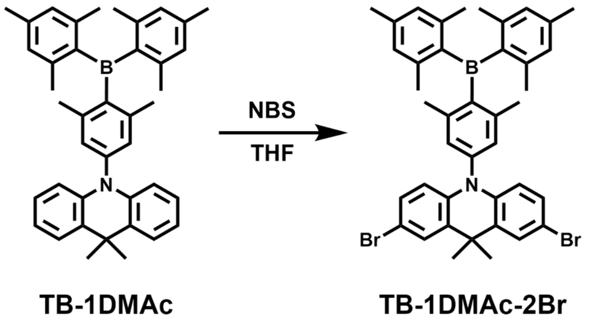

Scheme S2. Synthetic route of TB-1DMAc-2Br.

3. Supplementary Figures and Schemes

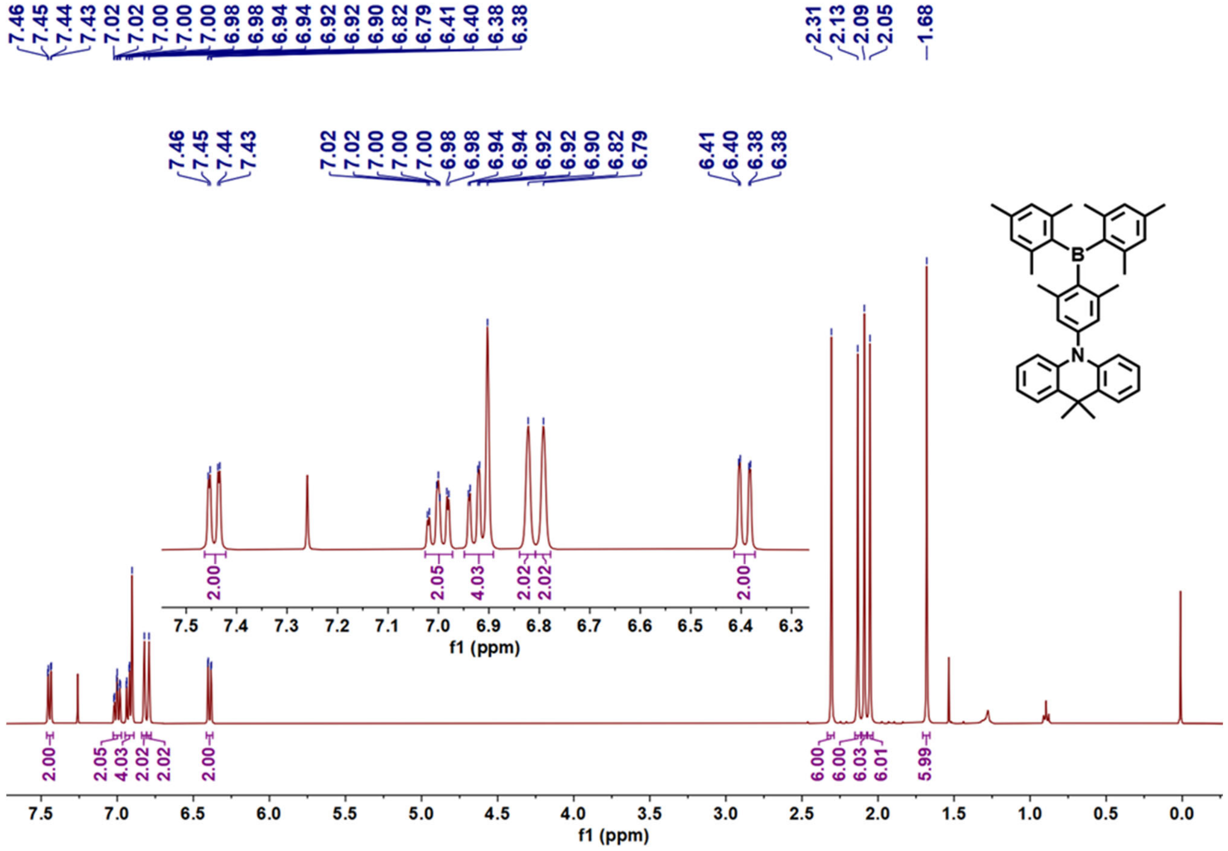

Figure S1. ^{1}H NMR spectrum of TB-1DMAc.

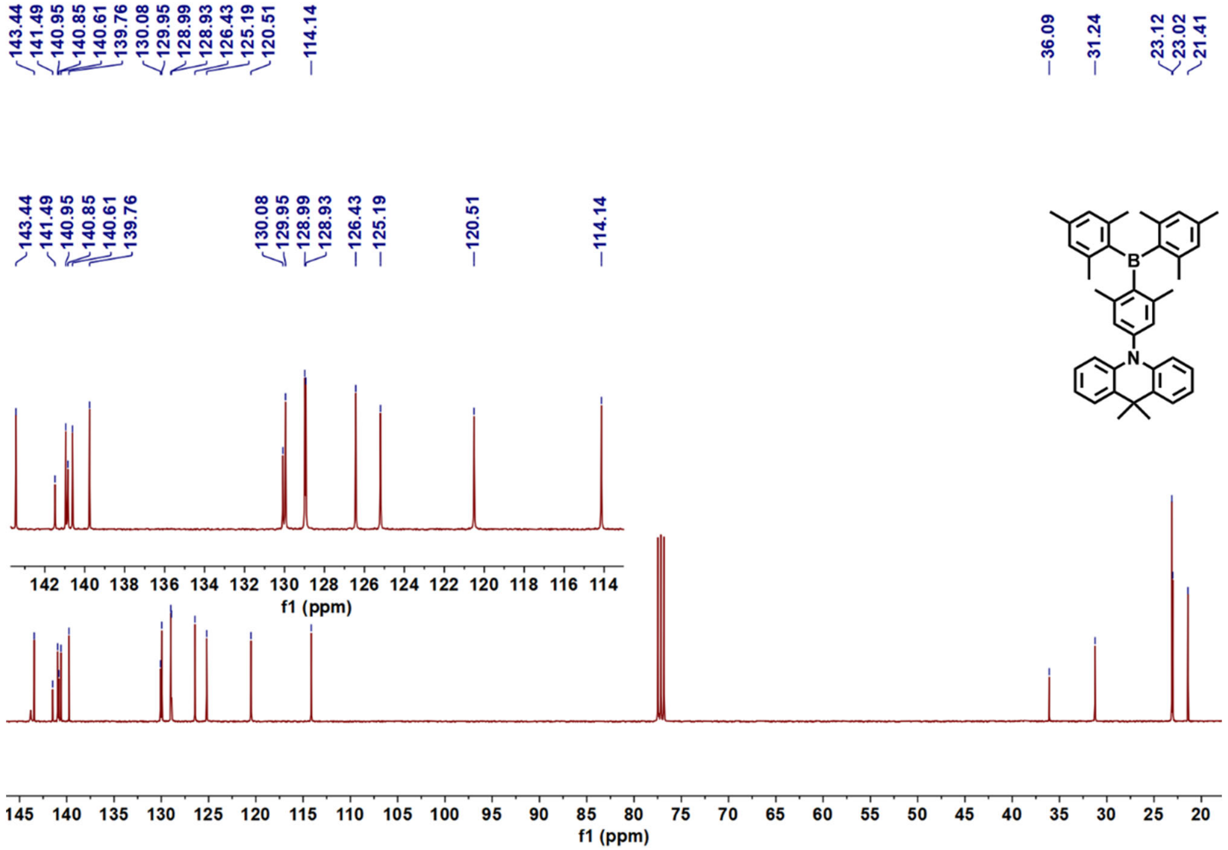

Figure S2. ^{13}C NMR spectrum of TB-1DMAc.

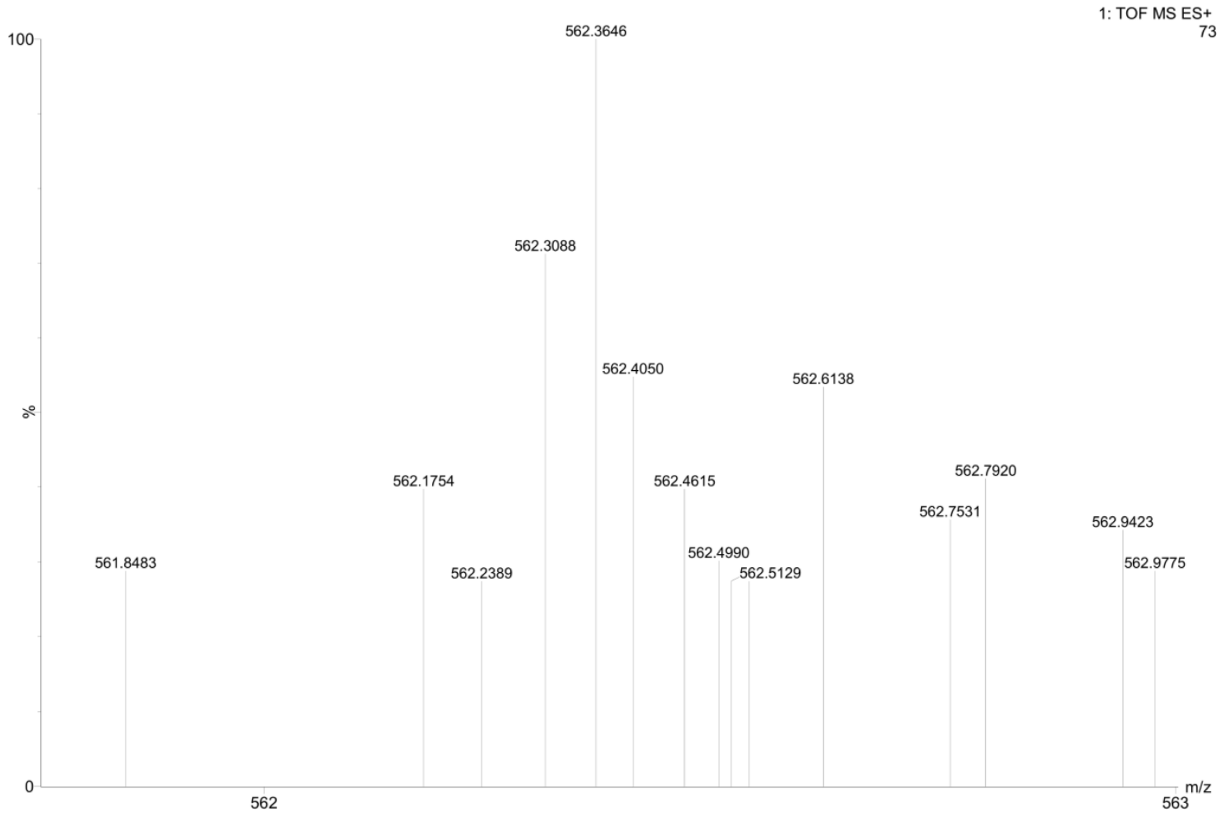

Figure S3. HRMS spectrum of TB-1DMAc.

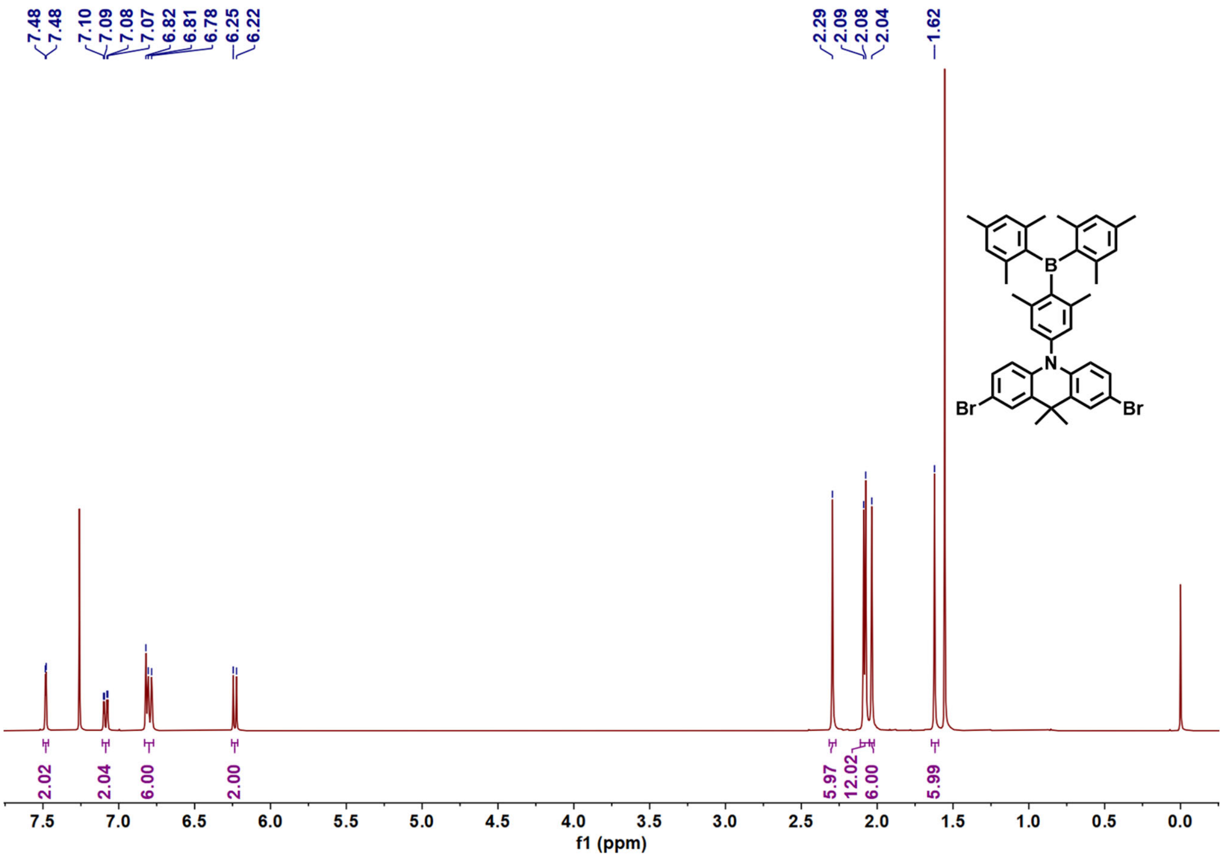

Figure S4. ^1H NMR spectrum of TB-1DMAc-2Br.

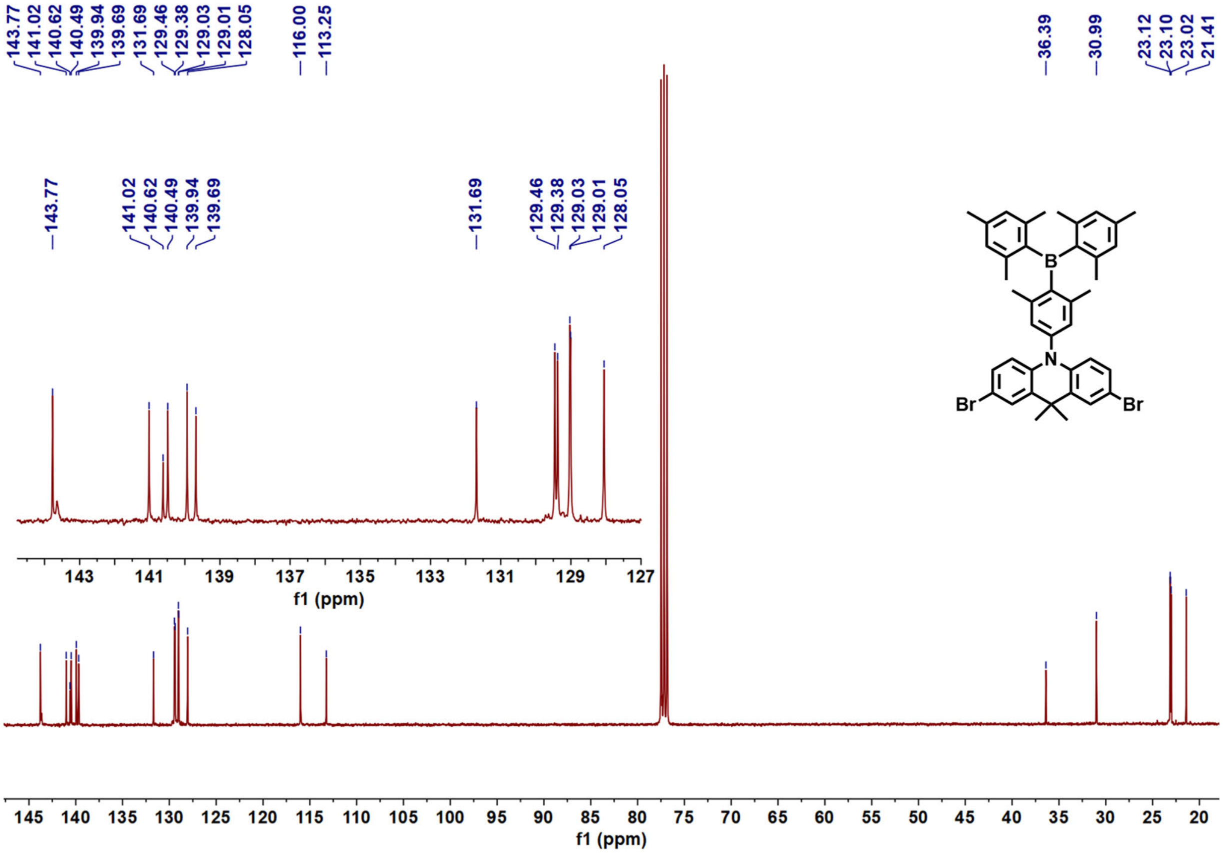

Figure S5. ^{13}C NMR spectrum of TB-1DMAc-2Br.

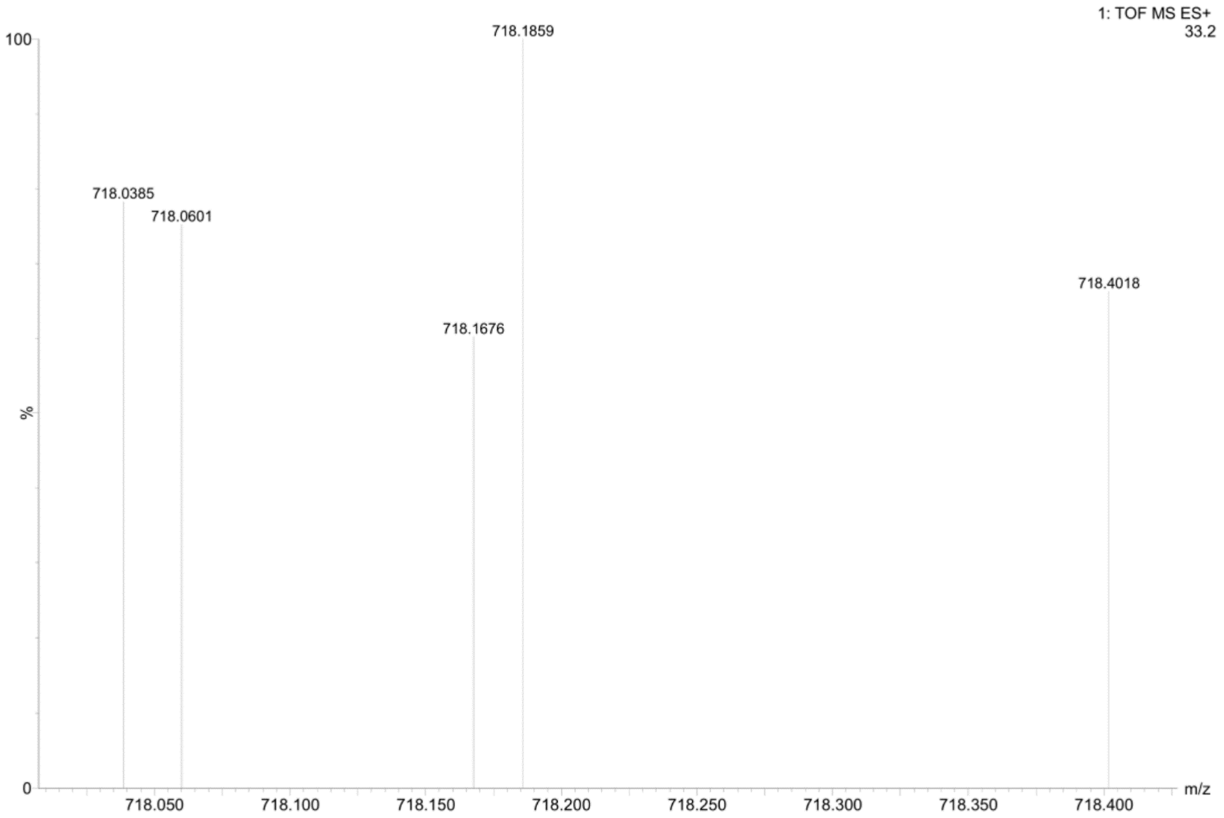

Figure S6. HRMS spectrum of TB-1DMAc-2Br.

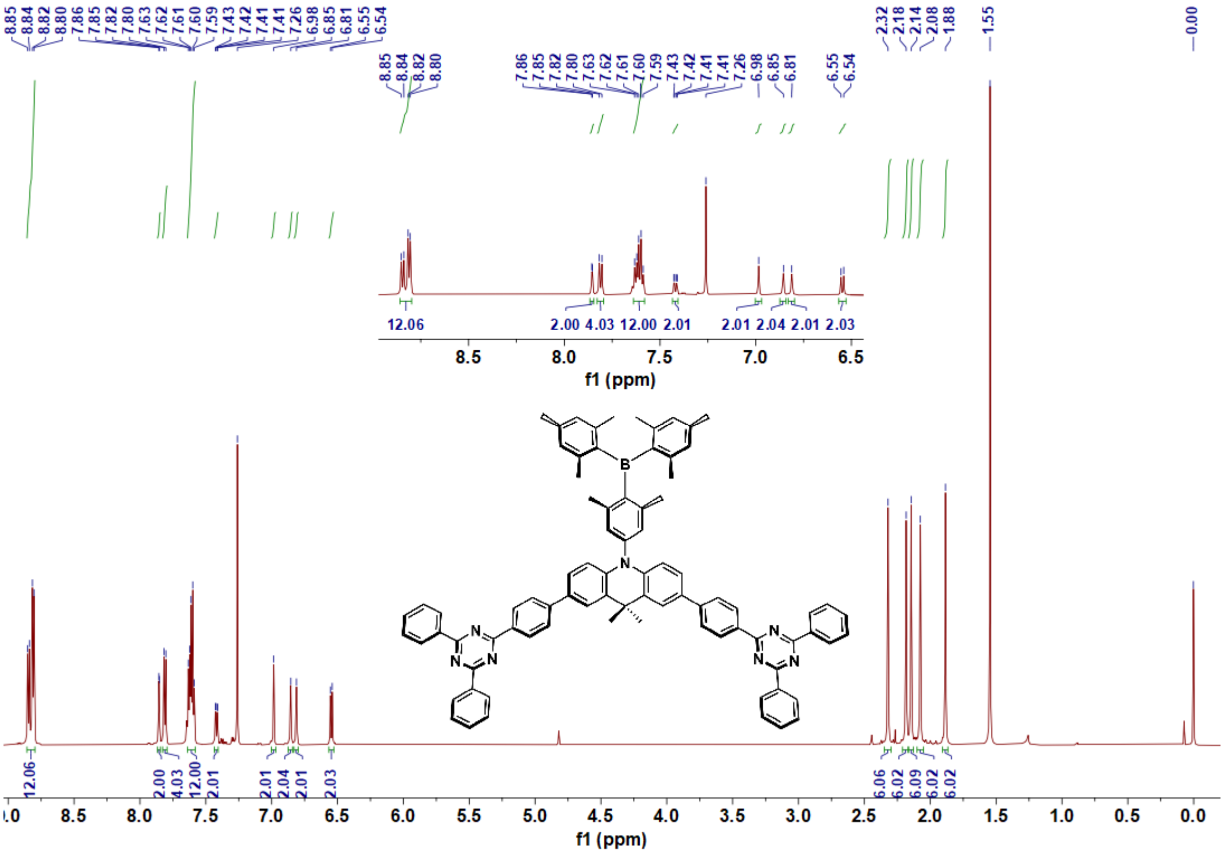

Figure S7. ^{1}H NMR spectrum of TB-1DMAc-2TRZ.

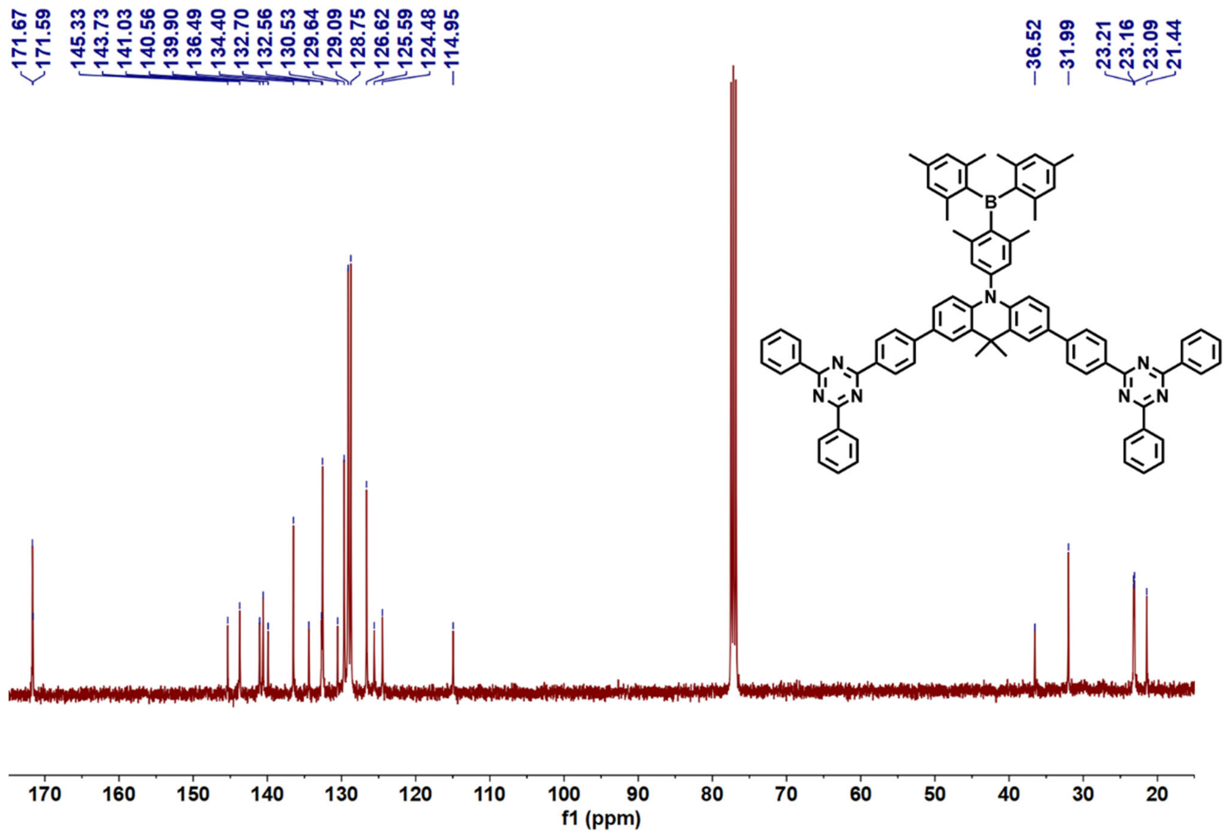

Figure S8. ^{13}C NMR spectrum of TB-1DMAc-2TRZ.

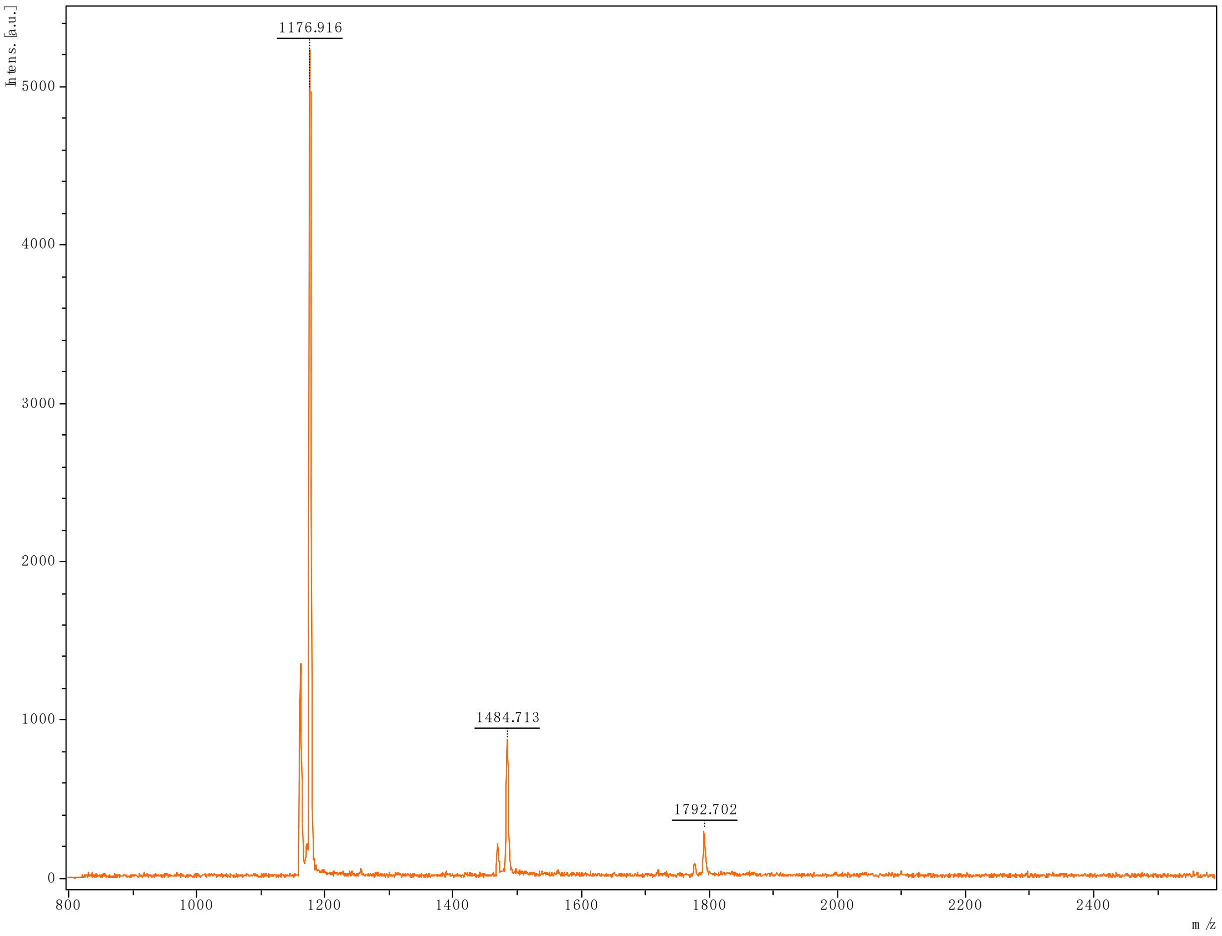

Figure S9. MS spectrum of TB-1DMAc-2TRZ.

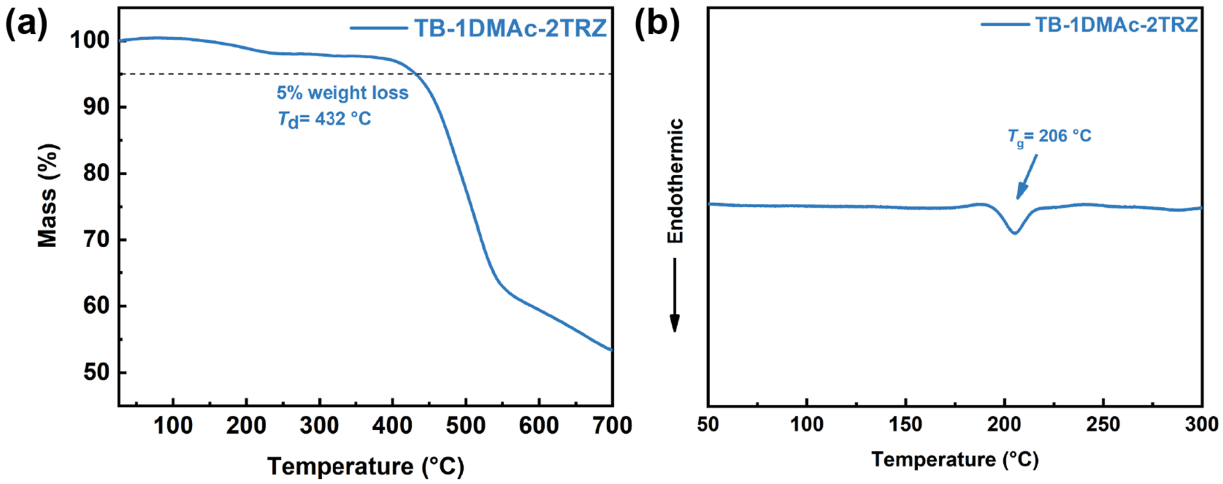

Figure S10. (a) TGA and (b) DSC of TB-1DMAc-2TRZ.

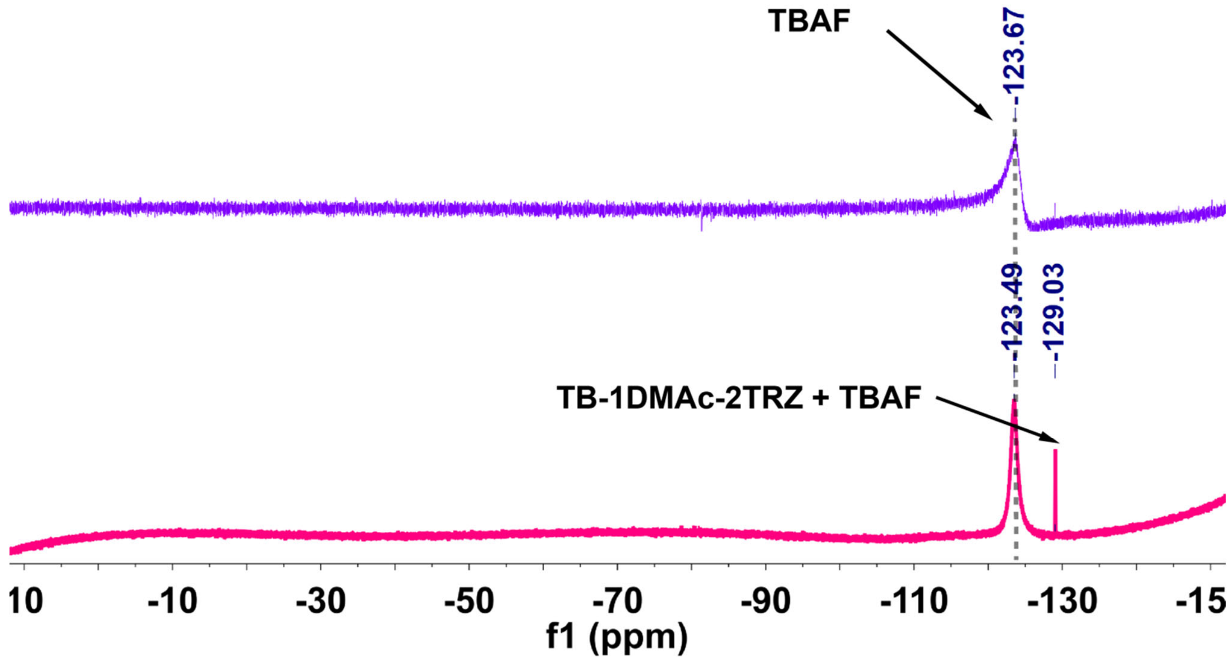

Figure S11. ^{19}F NMR spectra of TBAF and TB-1DMAc-2TRZ mixture after addition of TBAF.

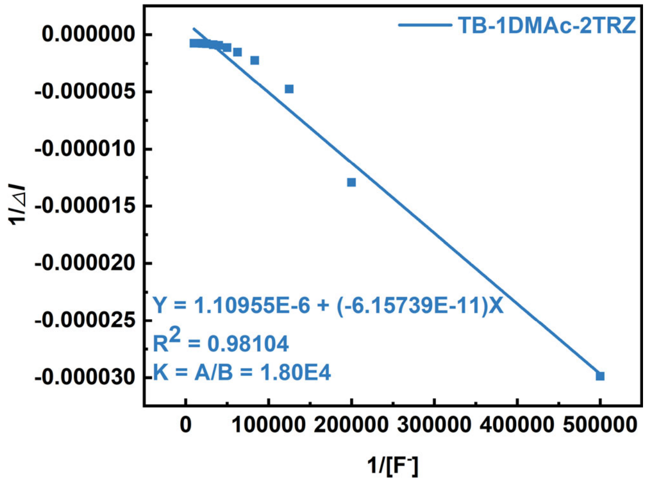

Figure S12. Benesi-Hildebrand plots for complexation of TB-1DMAc-2TRZ with F^-.

Table S1. Comparison of TB-1DMAc-2TRZ with other reported F^- detection probes.

Fluorescent Probe	Solvent	λ_{abs} (nm)	λ_{em} (nm)	DL (μM)	Ref.
CBT-NH	DMSO	400/470	626	0.86	[58]
PBT-NH	DMSO	400/480	505	4.25	

ICU	Dioxane	416	548	0.7	[1]
P-2	DMSO	425	576	0.034	[4]
Seleno-BODIPY	THF	451/615	688	0.043	[59]
YF-SZ-F	CH_3CN/PBS	430	695	0.402	[60]
TN-F	THF	345	419	0.63	[61]
BDBH	DMSO	532	613	0.27	[62]
1,8-naphthalimide-based	DMSO/H_2O	460	556	0.806	[63]
NAOZ	DMSO	462	533	0.927	[64]
NATZ	DMSO	462	533	2.47	
TB-1DMAc-2TRZ	THF	272/411	522	0.312	This work

References

1. Ali, R.; Dwivedi, S.K.; Mishra, H.; Misra, A. Imidazole-coumarin containing D-A type fluorescent probe: Synthesis photophysical properties and sensing behavior for F^- and CN^- anion. *Dyes Pigm.* **2020**, *175*, 108163.

4. Muthusamy, S.; Rajalakshmi, K.; Ahn, D.-H.; Kannan, P.; Zhu, D.; Nam, Y.-S.; Choi, K.Y.; Luo, Z.; Song, J.-W.; Xu, Y. Spontaneous detection of F^- and viscosity using a multifunctional tetraphenylethene-lepidine probe: Exploring environmental applications. *Food Chem.* **2025**, *466*, 142147.

55. Suzuki, N.; Suda, K.; Yokogawa, D.; Kitoh-Nishioka, H.; Irle, S.; Ando, A.; Abegão, L.M.G.; Kamada, K.; Fukazawa, A.; Yamaguchi, S. Near infrared two-photon-excited and -emissive dyes based on a strapped excited-state intramolecular proton-transfer (ESIPT) scaffold. *Chem. Sci.* **2018**, *9*, 2666-2673.

56. Lee, Y.H.; Lee, D.; Lee, T.; Lee, J.; Jung, J.; Yoo, S.; Lee, M.H. Impact of boryl acceptors in *para*-acridine-appended triarylboron emitters on blue thermally activated delayed fluorescence OLEDs.

Dyes Pigm. **2021**, *188*, 109224.

57. Buss, B.L.; Lim, C.H.; Miyake, G.M. Dimethyl dihydroacridines as photocatalysts in organocatalyzed atom transfer radical polymerization of acrylate monomers. *Angew. Chem., Int. Ed.* **2020**, *59*, 3209-3217.

58. Wu, J.; Lai, G.; Li, Z.; Lu, Y.; Leng, T.; Shen, Y.; Wang, C. Novel 2,1,3-benzothiadiazole derivatives used as selective fluorescent and colorimetric sensors for fluoride ion. *Dyes Pigm.* **2016**, *124*, 268-276.

59. Cugnasca, B.S.; Duarte, F.; Petrarca de Albuquerque, J.L.; Santos, H.M.; Luis Capelo-Martínez, J.; Lodeiro, C.; Dos Santos, A.A. Precision detection of cyanide, fluoride, and hydroxide ions using a new tetraseleno-BODIPY fluorescent sensor. *J. Photochem. Photobiol., A* **2024**, *457*, 115881.

60. Chen, J.; Yao, Y.; Pei, X.; Qu, M.; Zhang, J.; Hu, W.; Zhang, Y.; Wu, W.; Pei, S. A multifunctional near-infrared fluorescent probe based on benzothiazole structure for fluoride-ion detection. *Spectrochim. Acta, Part A* **2025**, *324*, 125009.

61. Zhou, M.-G.; Chen, L.; Zhou, J.; Zhong, X.; Yuan, M.-S. A fluorescence probe based on naphthalimide-functionalized triarylboron for fluoride ion detection. *Inorg. Chem. Commun.* **2024**, *170*, 113392.

62. Chen, X.; Liu, Y.-C.; Bai, J.; Fang, H.; Wu, F.-Y.; Xiao, Q. A “turn-on” fluorescent probe based on BODIPY dyes for highly selective detection of fluoride ions. *Dyes and Pigments* **2021**, *190*, 109347.

63. Xiao, L.; Ren, L.; Jing, X.; Li, Z.; Wu, S.; Guo, D. A selective naphthalimide-based colorimetric and fluorescent chemosensor for “naked-eye” detection of fluoride ion. *Inorg. Chim. Acta* **2020**, *500*, 119207.

64. Zhang, L.; Zhang, F.; Ding, L.; Gao, J. Reusable colorimetric and fluorescent chemosensors based on 1,8-naphthalimide derivatives for fluoride ion detection. *Spectrochim. Acta, Part A* **2020**, *237*, 118395.
